# High-Performance and Hysteresis-Free Perovskite Solar Cells Based on Rare-Earth-Doped SnO_2_ Mesoporous Scaffold

**DOI:** 10.34133/2019/4049793

**Published:** 2019-11-06

**Authors:** Qiyao Guo, Jihuai Wu, Yuqian Yang, Xuping Liu, Zhang Lan, Jianming Lin, Miaoliang Huang, Yuelin Wei, Jia Dong, Jinbiao Jia, Yunfang Huang

**Affiliations:** ^1^Engineering Research Center of Environment-Friendly Functional Materials, Ministry of Education, Institute of Materials Physical Chemistry, Huaqiao University, Xiamen 361021, China; ^2^School of Physics and Physical Engineering, Qufu Normal University, Qufu 273165, China; ^3^School of Chemical Engineering, Huaqiao University, Xiamen 361021, China

## Abstract

Tin oxide (SnO_2_), as electron transport material to substitute titanium oxide (TiO_2_) in perovskite solar cells (PSCs), has aroused wide interests. However, the performance of the PSCs based on SnO_2_ is still hard to compete with the TiO_2_-based devices. Herein, a novel strategy is designed to enhance the photovoltaic performance and long-term stability of PSCs by integrating rare-earth ions Ln^3+^ (Sc^3+^, Y^3+^, La^3+^) with SnO_2_ nanospheres as mesoporous scaffold. The doping of Ln promotes the formation of dense and large-sized perovskite crystals, which facilitate interfacial contact of electron transport layer/perovskite layer and improve charge transport dynamics. Ln dopant optimizes the energy level of perovskite layer, reduces the charge transport resistance, and mitigates the trap state density. As a result, the optimized mesoporous PSC achieves a champion power conversion efficiency (PCE) of 20.63% without hysteresis, while the undoped PSC obtains an efficiency of 19.01%. The investigation demonstrates that the rare-earth doping is low-cost and effective method to improve the photovoltaic performance of SnO_2_-based PSCs.

## 1. Introduction

As a new generation of thin film photovoltaic technology, organometallic halide perovskite solar cells (PSCs) have been attracting considerable interest owing to their high efficiencies, minor environmental impact, and facile solution processability [1–4]. Since the birth of first prototype in 2009 [5], the power conversion efficiency (PCE) of PSCs has undergone rapid increment from 3.8% to 23.48% (certified) during the past several years [6]. This outstanding progress is attributed to the unremitting efforts of researchers on optimizing chemical composition of perovskite and deposition processes [7–10], as well as perovskite prominent optoelectronic properties, such as bandgap adjustability [11, 12] and long carrier lifetime [13–15]. Interestingly, the emergence of perovskite solar cells originated from dye-sensitized solar cells (DSSCs). In turn, the development of perovskite solar cells promoted the research of DSSCs, especially in polymer electrolytes and flexible devices [16–22].

Titanium dioxide (TiO_2_) is a frequently used electron transport material for perovskite solar cells. However, TiO_2_ shows a lower electron mobility of (0.1~1.0 cm^2^ V^–1^ s^–1^), compared with conventional perovskite material (20~30 cm^2^ V^–1^ s^–1^), which causes insufficient charge carrier separation at the TiO_2_/perovskite interface [23–25]. In addition, the UV instability of TiO_2_ upon UV exposure triggers a rapid decrease in performance of PSCs via the degradation of the organic components in the PSC (especially for mesoporous TiO_2_-based PSCs) [26, 27]. Furthermore, high temperature processing (HTP) for TiO_2_ electron transport layer (ETL) is also unfavorable for the fabrication of low-cost PSCs. To overcome these issues, various metal oxides (i.e., SnO_2_, ZnO, WO_3_, In_2_O_3_, and SrTiO_3_) and fullerene are investigated as the substitute electron transport materials for TiO_2_ [28–32]. Among them, SnO_2_ has emerged as an especially promising candidate, owing to its low temperature processability, high optical transmittance in visible range, high electron mobility (100~200 cm^2^ V^–1^ s^–1^), less sensitive to UV radiation, and favorable energy level alignment to perovskite absorbers [33]. However, solution-processed planar SnO_2_ films suffer from temperature-dependent nonideal electron mobility at low annealing temperature or crack defect morphology brought by high annealing temperature (i.e., 500°C), which both lead to inferior contact and electron transport at the ETL/perovskite interfaces and then manifested in *J-V* hysteresis [34–36]. Fortunately, a thin mesoporous ETL can be designed to ameliorate these issues by improving perovskite coverage condition to get large perovskite grains [37–39]. On this basis, mesoporous SnO_2_ (m-SnO_2_) ETL has been highlighted for the industrial production of stable and efficient SnO_2_-based PSCs [40]. The PSCs embedded with full SnO_2_ blocking layer (bl)/mesoporous (mp) layer have obtained inspiring advances [41]. Higher PCEs of 13.1% and 17% were obtained employing HTP m-SnO_2_ and gallium-doped m-SnO_2_ by Roose et al. [40, 42]. Afterwards, Liu et al. and Yang et al. reported the PSCs with LTP 2D SnO_2_ nanosheet arrays and yttrium-doped SnO_2_ nanosheet arrays; the PCEs increased to 16.17% and 17.29% [34, 43]. Recently, Xiong et al. reported a high-stabilized PCE of 19.12% on the planar Mg-SnO_2_ PSC by using HTP m-SnO_2_ scaffold [36]. More recently, low-temperature processed (LTP) planar SnO_2_ PSCs have been achieved a PCE of 21.4% by using tin oxide precursors based on acetylacetonate [44].

In this work, monodisperse mesoporous SnO_2_ nanospheres with large surface area are synthesized under 300°C posttreatment and are used as mesoporous scaffold in PSCs to improve photovoltaic performance of devices by modifying perovskite coverage condition with large perovskite grains without damaging the surface morphology of SnO_2_ blocking layer. Moreover, rare-earth cations located in the third subgroup (Sc^3+^, Y^3+^, La^3+^) are introduced into the SnO_2_ mesoporous scaffold to reduce trap state density and transport resistance, enhance charge carrier concentration, and regulate energy level of SnO_2_, resulting in enhancement of photovoltaic parameters of the device. By optimizing the amount of Ln^3+^ dopants, the 3%-SNOY device exhibits a hysteresis-free and high-stabilized power conversion efficiency of 20.63%, superior to those reported previously for full SnO_2_ mesoporous structure PSCs. Furthermore, the UV stability is also investigated to illustrate the excellent long-term stability of these full SnO_2_-based PSCs.

## 2. Results and Discussion

### 2.1. Structure and Morphologies

**Figure 1:**
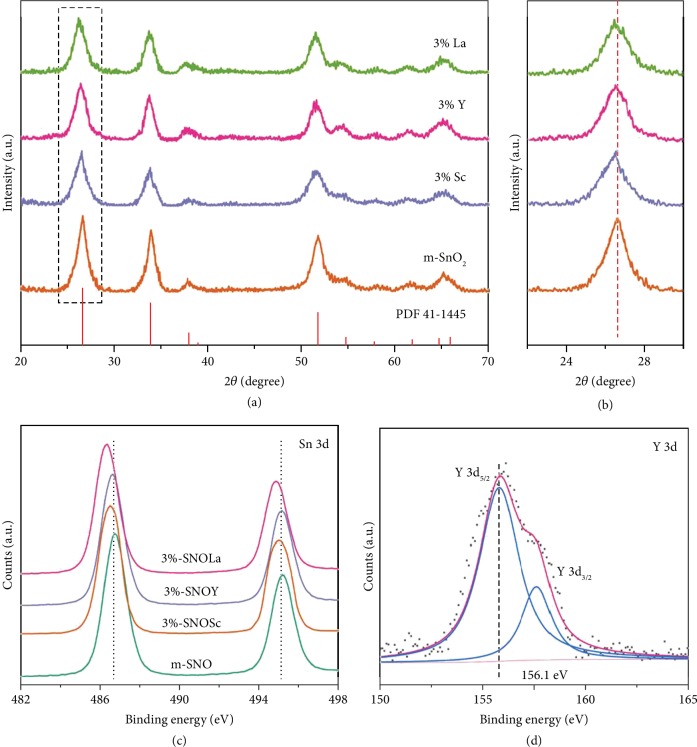
(a) XRD patterns of undoped m-SnO_2_ and 3% Ln^3+^ (Sc^3+^, Y^3+^, La^3+^)-doped m-SnO_2_. (b) Magnified XRD diffraction peaks for the selected region. (c), (d) XPS spectra of Sn3d and Y3d, respectively.

For research, SnO_2_ were synthesized with planar (p-SNO), mesoporous (m-SNO), and doped Ln in Ln^3+^/Sn^4+^ molar ratio (*x*%-SNOLn).  Figure 1(a) shows the X-ray diffraction pattern (XRD) of m-SnO_2_ and 3% Ln^3+^ (Sc^3+^, Y^3+^, La^3+^)-doped m-SnO_2_. All peaks are readily indexed to the tetragonal rutile phase of SnO_2_ (JCPDS card No. 41-1445); no additional peaks are observed, indicating that Ln^3+^ (Sc^3+^, Y^3+^, La^3+^) dopants do not change the phase structure of SnO_2_. The range (2*θ* = 24~28°) is magnified in Figure 1(b). The peaks for doped samples are slightly broader and shift to lower angels compared with the peak for undoped sample. This is due to the partially substitutional or interstitial incorporation of Ln^3+^ and the radius difference between Sc^3+^ (0.087 nm), Y^3+^ (0.1019 nm), La^3+^ (0.116 nm), and Sn^4+^ (0.069 nm), suggesting there is no distortion in the bulk SnO_2_ lattice due to the small amount of doping.

The X-ray photoelectron spectroscopy (XPS) scans for all samples, as shown in [Supplementary-material supplementary-material-1]–[Supplementary-material supplementary-material-1], highlight that no impurities are found in the spectra, suggesting that the Ln^3+^ ions are incorporated successfully into m-SnO_2_. From the high-resolution XPS spectra of Sn 3d (Figure 1(c)), undoped m-SnO_2_ shows two signal peaks at 486.7 and 495.2 eV, respectively, corresponding to Sn 3d 5/2 and Sn 3d 3/2 states of Sn^4+^. After doping, all Sn 3d peaks shift to lower binding energies, arising from the variations of the chemical environment for Sn^4+^. In detail, the different binding interactions between Ln-O and Sn-O lead to the charge transfer effect around Sn^4+^ species [45]. Besides, compared with the binding energy of Sc_2_O_3_ (401.9 eV), Y_2_O_3_ (156.6 eV), and Ln_2_O_3_ (835.2 eV) [46], the shift to lower binding energy with different degrees indicates that Ln^3+^ exists in a substitutional or interstitial bonding mode of Sn-O-Ln [47]. The narrow spectra of Sc 2p, Y 3d, and La 3d are manifested in Figure 1(d) and [Supplementary-material supplementary-material-1], [Supplementary-material supplementary-material-1]; all characteristic peaks can be assigned to +3 oxidation state of Ln [48, 49].

**Figure 2:**
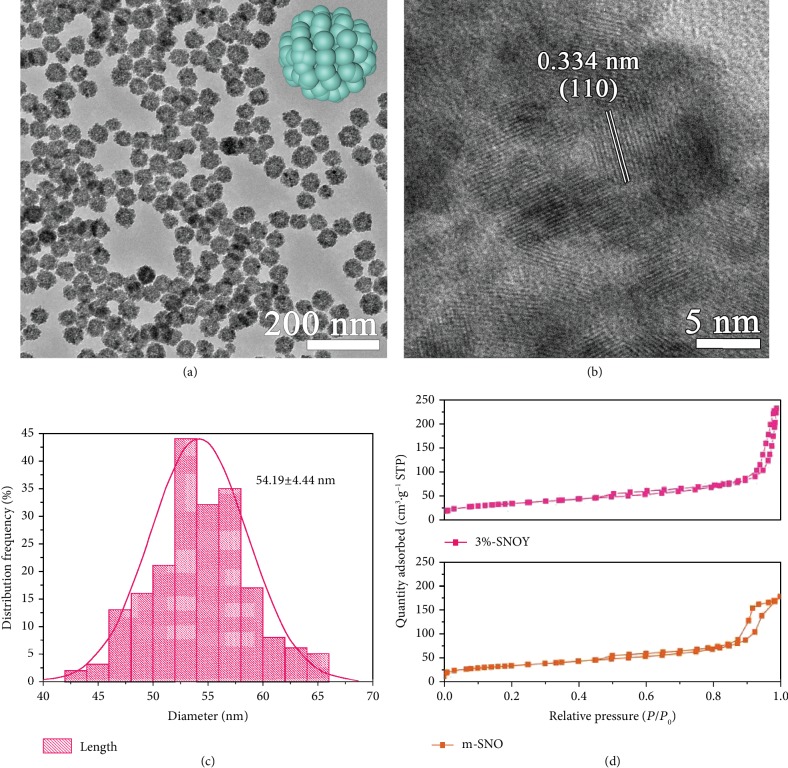
(a) TEM image of 3% Y-doped m-SnO_2_. (b) HRTEM image of 3% Y-doped m-SnO_2_. (c) Histogram of particle diameters from (b). (d) Nitrogen adsorption-desorption isotherms of the as-synthesized samples.

Figure 2(a) shows TEM image of 3% Y-doped m-SnO_2_, which mainly consists of monodisperse nanospheres with the average diameter about 54.19 ± 4.44 nm (Figure 2(c)). The enlarged TEM image shown in Figure 2(b) reveals that the 3% Y-doped m-SnO_2_ nanospheres have granular structure and coarse surface that consist of small SnO_2_ nanoparticles with a size of 6~8 nm, which endows the 3% Y-doped m-SnO_2_ nanospheres with high surface area. From Figure 2(b) and [Supplementary-material supplementary-material-1], the lattice spacing for m-SnO_2_ and 3% Ln^3+^-doped m-SnO_2_ is calculated as 0.334 nm, corresponding to the rutile SnO_2_ phase of (110) lattice planes, indicating that the Ln^3+^ dopants have no influence on the crystalline phase of m-SnO_2_, which is accorded with the XRD patterns (Figure 1(b)).

The pore width and the specific surface area of undoped m-SnO_2_ and 3% Y^3+^-doped m-SnO_2_, derived from nitrogen adsorption-desorption measurements, are shown in Figure 2(d) and [Supplementary-material supplementary-material-1]. All samples exhibit a type H3 hysteresis loop according to the Brunauer-Deming-Deming-Teller (BDDT) classification, indicating the presence of mesopores (2~50 nm) [50, 51]. For 3% Y^3+^-doped m-SnO_2_, the Barrett-Joyner-Halenda (BJH) desorption cumulative pore width is 13.8 nm, which is in agreement with the above BDDT classification. Compared with the pore width of undoped m-SnO_2_ sample (9.80 nm), an increment of 40.8% is achieved by 3% Y^3+^ dopant [34]. According to the BET method, the specific surface areas of undoped m-SnO_2_ and 3% Y^3+^-doped m-SnO_2_ are 120.2 and 130.0 m^2^ g^−1^, respectively. Based on the BET results, the pore feature (especially for pore width) of the doped sample is improved due to the addition of rare-earth ions, which is profitable for the penetration of the perovskite into the mesoporous scaffold and then the formation of well-aligned perovskite morphology.

Figures [Supplementary-material supplementary-material-1]–[Supplementary-material supplementary-material-1] show the FE-SEM images of m-SnO_2_ nanospheres with similar particle diameter for undoped and doped samples, indicating the doping hardly changes the morphology of particles; thus, the influence of morphology by doping on device performance can be excluded. However, the granular structure of m-SnO_2_ nanospheres collapses when the Ln^3+^ concentration is increased to 4% ([Supplementary-material supplementary-material-1], [Supplementary-material supplementary-material-1]) [34, 37]. Thus, a suitable Ln doping amount is crucial. In our experiment condition, the Ln optimal concentration is 3%.

**Figure 3:**
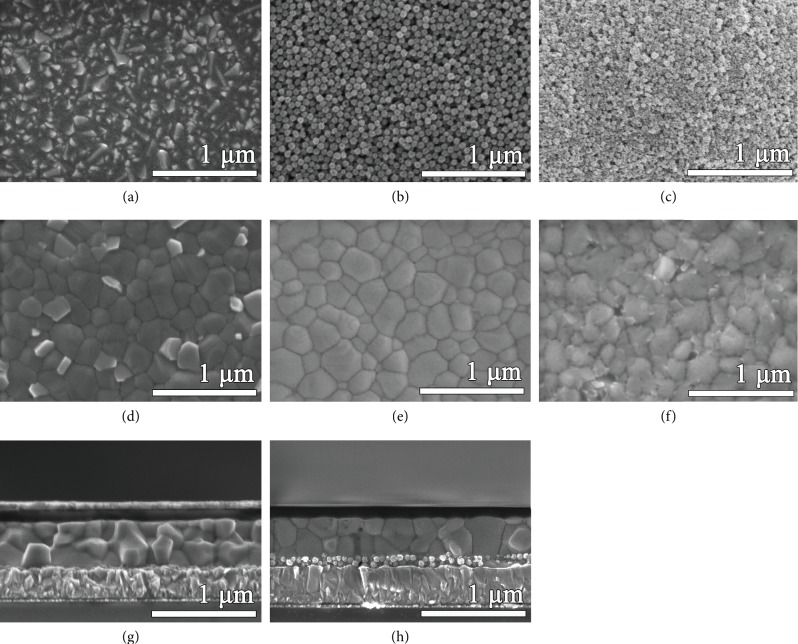
Top view FE-SEM images of (a) p-SNO, (b) 3%-SNOY, and (c) 4%-SNOY thin films deposited on FTO substrates. Top view FE-SEM images of perovskite films on (d) p-SNO, (e) 3%-SNOY, and (f) 4%-SNOY. Cross view FE-SEM image of the PSC based on (g) p-SNO and (h) 3%-SNOY mesoporous scaffold (annealed at 300°C).

Figure 3 shows the FE-SEM images of SnO_2_ films (annealed at 300°C), perovskite films, and the devices. [Supplementary-material supplementary-material-1] shows the corresponding FE-SEM images annealed at 180°C. In Figure 3(a) and [Supplementary-material supplementary-material-1], the morphologies of the p-SNO annealed at 300°C and 180°C are similar, indicating that the p-SNO films are thermal stable. From Figure 3(b), 3%-SNOY thin film consists of many monodisperse and well-aligned nanospheres, which increases contact area between m-SnO_2_ scaffold/perovskite layer and results in the improvement of *J*_SC_ and FF. From Figures 3(d)–3(f), 3%-SNOY perovskite film presents uniform, smooth-surface, and larger grains compared with that of the p-SNO perovskite film, while heavily Ln^3+^-doped m-SnO_2_ (4%-SNOY) (Figure 3(c)) leads to a rough and pin hole perovskite surface, which may have a detrimental effect on device performance. Compared with the cross-sectional SEM image of Figures 3(g) and 3(h), the SNOY-based perovskite film shows dense, large perovskite particles and thickness about 400 nm, proving that the perovskite is well crystallized in the 3%-SNOY scaffold.

### 2.2. Energy Band Structure

**Figure 4:**
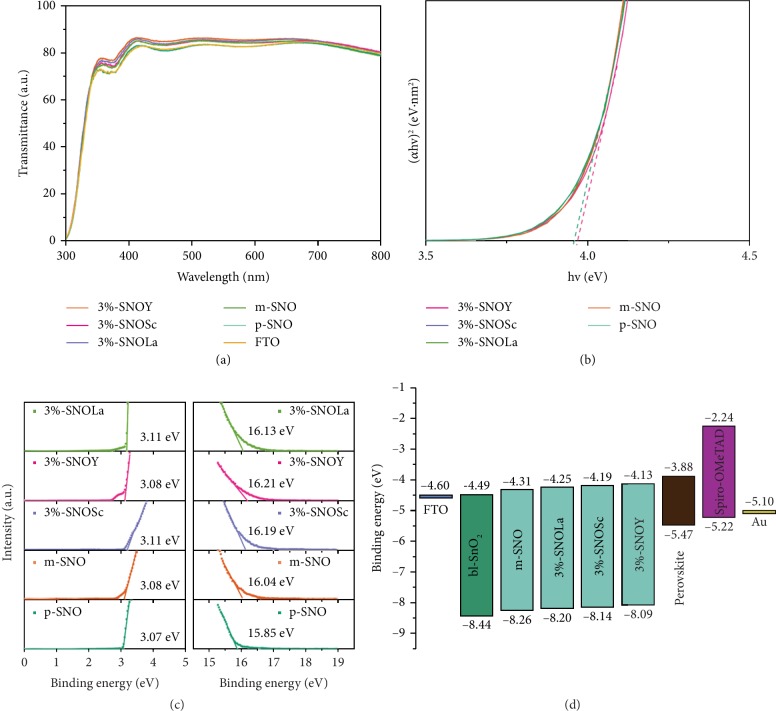
(a) Transmittance spectra of films. (b) Tauc plots corresponding to the transmission spectra. (c) Ultraviolet photoelectron spectroscopy (UPS) spectra of films in the on-set (left) and the cutoff (right) region. (d) Experimentally determined energy level diagrams (relative to the vacuum level) of different component layers in the PSC devices.

High optical transmission facilitates efficient utilization of sunlight and leads to an improved *J*_SC_ and PCE for PSCs.  Figure 4(a) shows the optical transmission of the films in the order: 3% − SNOY > 3% − SNOSc > 3% − SNOLa > m − SNO > p − SNO, which agrees with the photovoltaic performance of the devices. From Figure 4(b), the optical band edges (*E*_g_) are calculated according to the formula: (*αhν*)^*n*^ = *A*(*hν* − *E*_g_) (*n* = 1/2), and results are listed [Supplementary-material supplementary-material-1]. Various samples have almost the same *E*_g_ values (3.95 eV), indicating that the low amount of Ln doping does not affect *E*_g_ of the film.

Based on the equation of (Fermi level) *E*_F_ = *E*_cutoff_(cutoff binding energy) − 21.2 eV (emission energy from He irradiation) and Figure 4(c), *E*_F_ of samples are calculated and the results are listed in [Supplementary-material supplementary-material-1]. The gradual upward shift of the *E*_F_ confirms the improvement in carrier concentration crosschecked by conductivity and Mott-Schottky measurements mentioned after [43, 52]. According to *E*_VB_ = *E*_F_ − *E*_on−set_ (on-set binding energy) and *E*_CB_ = *E*_VB_ + *E*_g_, the valence band positions (*E*_VB_) and the conduction band (*E*_CB_) are calculated and expressed in Figure 4(d) and [Supplementary-material supplementary-material-1]. SnO_2_ mesoporous scaffold plays a functional bridging role between SnO_2_ bl and perovskite layer; the increase of *E*_CB_ values of p-SNO, m-SNO to SNOLn films is beneficial for the charge carrier extraction, which the results are good coincidence with the *J-V* measurements. Meanwhile, the enhancement of *V*_OC_ can be attributed to conduction band upward shift, influenced by the *E*_CB_ and *E*_VB_ of ETL and hole transport layer (HTL). According to the above discussions, the enhancement in photovoltaic parameters is ascribed to the optimization of morphology of perovskite film and charge transport dynamics.

### 2.3. Photoelectrochemical Properties

The current-voltage (*I-V*) curves of the FTO/ETL/Au devices were measured in dark and are shown in [Supplementary-material supplementary-material-1]. The conductivities of samples are calculated as 1.15 × 10^−5^ (m-SNO) to 2.76 × 10^−5^ S cm^−1^ (3%-SNOY), according to *σ* = *d*/*AR*, where *d* is the film thickness, *A* is the film area, and *R* is the film resistance. The enhanced conductivity indicates the passivation of charge trap states by Ln^3+^ doping and facilitates the charge extraction for improving *J*_SC_ and FF values.

[Supplementary-material supplementary-material-1] shows Mott-Schottky (M-S) analysis of samples; all films show positive slope for *n*-type. The flat band potential *V*_fb_, calculated from the intersection of the linear region with the *X*-axis, is 0.49 V (m-SNO), 0.35 V (3%-SNOSc), 0.29 V (3%-SNOY), and 0.41 V (3%-SNOLa) vs. Ag/AgCl (equivalent to 0.69, 0.55, 0.49, and 0.61 V vs. NHE). Fermi level (*E*_F_) is calculated according to the empirical formula *E*_F_ = −(*V*_fb_ + 4.5) eV by assuming the energy level of normal hydrogen electrode as −4.5 eV [53]. Thus, the *E*_F_ values of the films are −5.19 eV (m-SNO), −5.05 eV (3%-SNOSc), −4.99 eV (3%-SNOY), and −5.11 eV (3%-SNOLa), which are consistent with the UPS results. The M-S curves can be used to analyze the number of free electrons (*N*_e_), which is inversely proportional to the straight line slope of the M-S plot using the equation: slope = 2/*εε*_0_*A*^2^*qN*_e_, where *ε* is the relative dielectric constant for SnO_2_, *ε*_0_ is the vacuum permittivity, *A* is the sample area, and *q* is the elementary charge [43, 54, 55]. The slope of the 3%-SNOY film (2.10 × 10^16^) is much smaller than that of m-SNO film (10.22 × 10^16^), suggesting a considerable increment of *N*_e_. Simultaneously, it also confirms that Ln^3+^ ions are incorporated interstitially into SnO_2_ as *p*-type dopants, which make the increment of *N*_e_ [43].

To further elucidate the effect of Ln^3+^ doping on hysteretic behavior of the device, we carried out steady-state photoluminescence (PL) quenching experiment and time-resolved photoluminescence (TRPL) intensity decay measurement. As presented in [Supplementary-material supplementary-material-1], the samples with mesoporous scaffold show a faster electron quenching efficiency than the p-SNO film. According to the result, 3%-SNOY shows the fastest quenching rate, indicating an efficient electron transport and charge extraction at the interface of perovskite/SnO_2_, which can prevent the accumulation of redundant capacitive charge that leads to hysteresis. [Supplementary-material supplementary-material-1] and [Supplementary-material supplementary-material-1] show the TRPL decays of samples; the curves were fitted with a two-component exponential decay function *I* = *A*_1_*e*^−(*t* − *t*0)/*τ*1^ + *A*_2_*e*^−(*t* − *t*0)/*τ*2^, where *τ*_1_ refers to the faster component of trap-mediated nonradiative recombination and *τ*_2_ is the slower component correlated to radiative recombination. The average PL decay times (*τ*_ave_) can be calculated with the formula *τ*_ave_ = (*A*_1_*τ*_1_^2^ + *A*_2_*τ*_2_^2^)/(*A*_1_*τ*_1_ + *A*_2_*τ*_2_), in which *A*_1_ and *A*_2_ represent the decay amplitudes. The fitted result for the 3%-SNOY film delivered a much faster PL decay rate (58.89 ns) than that of the p-SNO film (171.35 ns), validating the trap-assisted recombination is partly eliminated, which is beneficial for improving PCE output.

[Supplementary-material supplementary-material-1] illustrates the Nyquist plots of PSCs devices; the intercept point on the real axis in the high-frequency range is the series resistance (*R*_s_), the semicircle in the high-frequency range is the charge-transfer resistance (*R*_ct_) between at HTL/Au interface, and the value in low-frequency range is the recombination resistance (*R*_rec_) at mesoporous/perovskite layer interface. The measured resistances are listed in [Supplementary-material supplementary-material-1], the *R*_rec_ trend is in good coincidence with the *J-V* measurements, and the improvement of FF can be attributed to the optimization of charge-transfer resistance of devices owing to Ln^3+^ doping.

### 2.4. Photovoltaic Performance

**Figure 5:**
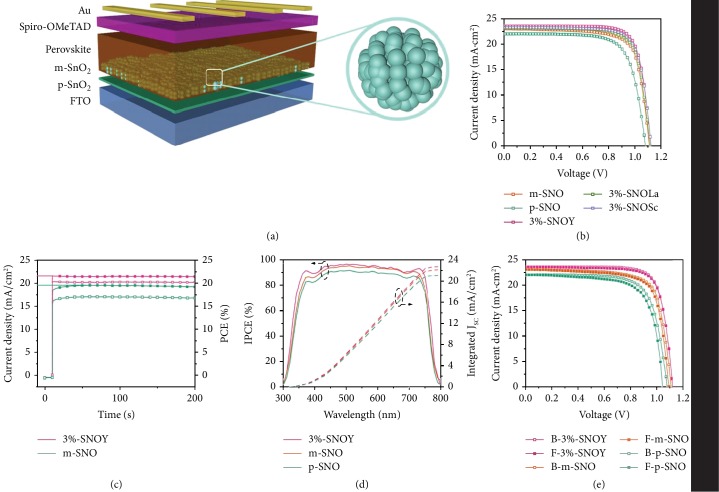
(a) Schematic device structure. (b) *J-V* curves of the PSCs with different SnO_2_. (c) Steady-state output of *J*_SC_ and PCE for m-SNO and 3%-SNOY devices. (d) IPCE spectra of the PSCs with different SnO_2_. (e) *J-V* curves of the PSCs with different SnO_2_ under backward and forward scanning.

Figure 5(a) shows the structure diagram of fabricated PSCs. Figure 5(b) shows the characteristic photocurrent density-voltage curves (*J-V*) curves of the optimized p-SNO, m-SNO, and 3%-SNOLn (Sc^3+^, Y^3+^, La^3+^) devices; the relative photovoltaic data are given in [Supplementary-material supplementary-material-1]. Impressively, compared to the p-SNO device, m-SNO cell shows an improved PCE of 19.01%, indicating a 10.46% enhancement in PCE (19.01% vs. 17.21%), which attributes to efficient electron transport brought by dense, large size, and vertical distribution of perovskite (Figure 3(h)) and improved interfacial connection between SnO_2_ scaffold and perovskite. We note that PCE reach a highest value of 20.63% for 3%-SNOY device, owing to the morphology optimization of perovskite layer, the adjustment of energy level alignment with perovskite layer, and the improvement of charge transport dynamics by Y^3+^ doping. The performance of PSCs is further improved owing to Ln ion doping, obeying an order of 3% − SNOY > 3% − SNOSc > 3% − SNOLa > m − SNO > p − SNO, yielding a champion PCE as high as 20.63% for 3%-SNOY tailored PSC. The photovoltaic performance of the 3%-SNOY device is higher than that of state-of-the-art PSCs based on mesoporous SnO_2_ scaffold ([Supplementary-material supplementary-material-1]).

Figures [Supplementary-material supplementary-material-1]–[Supplementary-material supplementary-material-1] and [Supplementary-material supplementary-material-1] show *J-V* curves and photovoltaic parameters of the PSCs based on m-SNO layer doped with different Ln ion concentrations. To ensure the reliability and repeatability of data, average photovoltaic parameters of the each PSC device were obtained from 20 devices. As mentioned above, excessive dopants are detrimental to the device performance either by the hoist of conduction band leading to an inefficient electron injection, in which case *V*_OC_ keeps increasing, but *J*_SC_ decreases, or the dopants induce trap states, leading to a decrease of all device parameters [43]. In our case, both the trap state density increase induced by heavily Ln^3+^-doped m-SnO_2_ (4%-Sc^3+^, Y^3+^, La^3+^) and the dreadful recombination occurring at the interface of mesoporous scaffold/perovskite induced by the scaffold breakdown drag the parameters of PSC devices. The reproducibility statistics of 20 devices shown in Figures [Supplementary-material supplementary-material-1]–[Supplementary-material supplementary-material-1] and [Supplementary-material supplementary-material-1] further confirm the better photovoltaic data for the 3%-SNOLn devices than the p-SNO devices. Apparently, the 3%-SNOY device exhibits the highest average PCE value (20.26 ± 0.116%), and average *J*_SC_, *V*_OC_, and FF values of 3%-SNOY device are the highest among all devices. The reliability and repeatability tests distinctly demonstrate the champion performance of the 3%-SNOY devices.

Figure 5(c) shows the steady-state output of *J*_SC_ and PCE for m-SNO and 3%-SNOY devices by tracking maximum power point (MPP) at a bias voltage 0.87 V and 0.94 V as shown in Figure 5(b). The p-SNO and 3%-SNOY devices yield stabilized PCE of 16.79% and 20.28%, respectively, which are comparable to the PCE obtained from the fresh *J-V* curves. Figure 5(d) demonstrates incident photon-to-current conversion efficiency (IPCE) spectra of p-SNO, m-SNO, and 3%-SNOY champion devices to verify the validity of the power output. The 3%-SNOY device exhibits the highest integrated *J*_SC_ value of 22.75 mA cm^−2^ compared to m-SNO (22.18 mA cm^−2^) and p-SNO (21.15 mA cm^−2^), concluding an efficient charge injection and well energy level alignment for 3%-SNOY film. In our case, the *J*_SC_ values derived from IPCE spectra are undervalued compared with that obtained from the corresponding *J-V* curves (Figure 5(b)), which is acceptable due to the spectral mismatch of the solar simulator and the theoretical AM 1.5G spectrum as reported before [56].

Figure 5(e) shows the *J-V* curves of the devices under backward (B 2 V ⟶ −0.2 V) and forward (F −0.2 V ⟶ 2 V) scanning, and the related data are summarized in [Supplementary-material supplementary-material-1]. Apparently, p-SNO device shows a mild hysteresis while 3%-SNOY device embodies a character of hysteresis-free. It denotes that the introduction of rare-earth ions improves charge carrier transport dynamics and suppresses the charge accumulation at the interfaces of SnO_2_ bl/m-SnO_2_ and m-SnO_2_/perovskite layers.

Figures [Supplementary-material supplementary-material-1] and [Supplementary-material supplementary-material-1] show the dependence of *J*_SC_ and *V*_OC_ on light intensity of the PSCs. The power law dependence of *J*_SC_ on the illumination intensity is generally expressed as *J*_SC_ ∝ *I*^*α*^, where *α* is the exponential factor related to bimolecular recombination [56]. The *α* value of 3%-SNOY (0.983) device is closer to 1 than that of m-SNO (0.973) and p-SNO (0.953) devices, suggesting a reduction of the bimolecular radiative recombination in the Ln^3+^ ion-doped devices due to more effective carrier transportation through the interfaces of SnO_2_ bl/m-SnO_2_/perovskite layers.

As shown in [Supplementary-material supplementary-material-1], the trap-assisted Shockley-Read-Hall recombination associated with trap state density is significantly suppressed due to the Ln^3+^ dopants determined as *V*_OC_ = nkTln(*I*)/q + constant [54], where *n* is an ideal factor related to monomolecular recombination, *k* represents the Boltzmann constant, and *T* is the absolute temperature. The slope of the plot (*n*) for the 3%-SNOY device is lower (1.32 kT/q) than that of the m-SNO (1.48 kT/q) and p-SNO devices (1.86 kT/q), indicating that the fewer monomolecular recombination in the 3%-SNOY device by Ln^3+^ doping, which is consistent with the [Supplementary-material supplementary-material-1].

[Supplementary-material supplementary-material-1] shows the dark *J*-*V* curves of PSCs, which is correlated to the leakage current from carrier recombination in the devices. According to the equation, *V*_OC_ = nkTln(*J*_SC_/*J*_0_)/q, where *J*_SC_ and *J*_0_ are the photogenerated and dark saturation current densities, respectively [57]. The value of *J*_0_ for the 3%-SNOY device is calculated as 8.41 × 10^−11^ mA cm^−2^, which is five orders of magnitude smaller than that of the p-SNO device (1.32 × 10^−6^ mA cm^−2^). While all three devices exhibit approximate output current, implying a higher rectification ratio and a greatly restrained leakage current induced by charge recombination for the device Ln^3+^ doped.

[Supplementary-material supplementary-material-1] illustrates the dark *J-V* curves of the electron-only devices with a structure of FTO/SnO_2_/perovskite/PCBM/Au. Apparently, the trap-filled limit voltages (*V*_TFL_) for 3%-SNOY device show a decreased trend in comparison with the other two devices, which is proportional to trap state density (*n*_trap_) according to *V*_TFL_ = *qn*_trap_*L*^2^/2*εε*_0_, where *L* is the thickness of the electron-only device, *ε* is the relative dielectric constant for SnO_2_, and *ε*_0_ is the vacuum permittivity [58]. These results provide strong evidences that the trap defects are healed significantly owing to Ln^3+^ ion doping, crosschecking the aforementioned conclusion.

### 2.5. Stability

**Figure 6:**
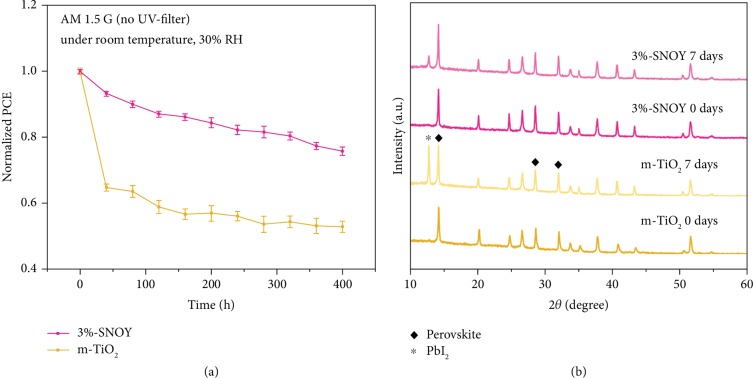
(a) Normalized PCE change with time for m-TiO_2_ and 3%-SNOY devices without encapsulation under simulated solar light illumination for 400 h. (b) XRD patterns of m-TiO_2_ and 3%-SNOY perovskite films in ambient condition (RH ~30%) before and after simulated solar light illumination for 7 days.

The long-term stability of perovskite solar cells should consider both ultraviolet light and humidity sensitivities [39, 59].  Figure 6(a) shows the normalized PCE vs. time of the devices without encapsulation under simulated solar light illumination for 400 h in 30% RH ambient condition. The m-TiO_2_ PSCs manifest a dramatic degradation to 64.8% of the highest PCE in the first 40 h arising from the photocatalytic properties of TiO_2_ degrading perovskite and then followed by a plain to 53.2% of the highest PCE originating from that the defects in TiO_2_ can be passivated by the adsorption of atmospheric oxygen [39]. While the 3%-SNOY devices decrease steadily at nearly 75.8% of the highest PCE under the same testing conditions, exhibiting remarkablely improved UV stability. [Supplementary-material supplementary-material-1] shows the change of average PCE values of 3%-SNOY and m-TiO_2_ devices with time, presenting similar results as Figure 6(a).

After exposure of the perovskite based on m-TiO_2_ to AM1.5 illumination under 30% RH ambient condition for one week shown in Figure 6(b), a new sharp diffraction peak at 12.7° corresponding to the PbI_2_ (001) lattice plane is observed in the XRD pattern, arising from the decomposition of the aged perovskite film caused by UV light and ambient humidity. The PbI_2_ would block the charge transport, resulting in a decrease of PCE. Notably, the intensity of PbI_2_ diffraction peak for the 3%-SNOY-based perovskite film aged in the same situations is very less, implying a much lower degree of decomposition, which is due to the passivation effect on defects and UV light stability by rare-earth ion doping.

## 3. Conclusions

In conclusion, rare-earth Ln-doped monodisperse SnO_2_ nanospheres with specific surface area of 130.0 m^2^ g^−1^ are successfully synthesized by a solution-phase route. The doped SnO_2_ nanospheres are used as scaffold to fabricate mesoporous perovskite solar cells. The observation of morphology, microstructure characterization, energy band analysis, photoelectric property investigation, and photovoltaic performance measurement indicate that the doping of rare-earth Ln ions promotes the formation of dense, even and large perovskite crystals, which facilitate better interfacial contacts of electron transport layer/perovskite layer. On the other hand, Ln dopants optimize the energy level of electron transport layer and reduce the resistance and charge trap states, resulting in an efficient electron transport and charge extraction. As a result, the Y^3+^ (3%)-doped mesoporous SnO_2_-based PSC achieved a champion efficiency of 20.63% with hysteresis-free, while the planar and mesoporous SnO_2_-based PSCs obtain efficiency of 17.21% and 19.01%, respectively. This investigation demonstrates a novel strategy for developing efficient and low-cost full SnO_2_-based PSCs.

## 4. Materials and Methods

### 4.1. Materials

Formamidinium iodide (FAI, 99.5%), methylammonium iodide (MAI, 99.5%), and methylammonium bromide (MABr, 99.5%) were obtained from Xi'an Polymer Light Technology Corp, China. Lead iodide (PbI_2_, 99.99%), lead bromide (PbBr_2_, 99%), and cesium iodide (CsI, 99%) were purchased from TCI. Bis(trifluoromethane) sulfonimide lithium salt (Li-TFSI, 99.95%), anhydrous dimethyl sulfoxide (DMSO, 99.9%), anhydrous *N*,*N*-dimethylformamide (DMF, 99.8%), 4-tert-butylpyridine (98%), anhydrous chlorobenzene (CB, 99.8%), anhydrous acetonitrile (99.8%), anhydrous 1-butanol (99.8%), and SnCl_2_·2H_2_O (99.995%) were received from Sigma-Aldrich. K_2_SnO_3_·3H_2_O (99.5%), urea (99.995%), YCl_3_·6H_2_O (99.99%), LaCl_3_·6H_2_O (99.99%), ScCl_3_·6H_2_O (99.9%), and ethylene glycol (EG, 99%) were obtained from Aladdin. All the chemicals were used as received without further purification. Deionized water (resistivity > 18 M*Ω*) was obtained through a Millipore water purification system. Prepatterned fluorine-doped tin oxide-coated (FTO) substrates with a sheet resistance of 14 *Ω* sq^−1^ were purchased from Pilkington.

### 4.2. Preparation of SnO_2_ and Ln-Doped Solutions

SnO_2_ solution was prepared by dissolving SnCl_2_·2H_2_O in 1-butanol in the concentration of 0.1 M. Monodisperse SnO_2_ nanospheres were synthesized by a solution-phase route [50]. In a typical procedure, 8 mL of aqueous solution containing LnCl_3_ (Sc^3+^, Y^3+^, La^3+^) and K_2_SnO_3_ with different Ln^3+^/Sn^4+^ molar ratios (0~4%) mixed with 15 mL of EG was added into a 50 mL Teflon-lined autoclave and maintained at 170°C for 13 h. The air-cooled precipitation was washed thoroughly with deionized water for removal of K^+^ and organic residue followed by a centrifugation and then diluted with deionized water in the concentration of 0.1 g mL^−1^ prior to use.

### 4.3. Fabrication of Perovskite Solar Cells

Laser-patterned FTO glass with size of 1.5 × 1.5 cm^2^ was cleaned by detergent and sonicating in isopropanol, acetone, deionized water, and ethanol and finally treated with UV ozone for 30 min. SnO_2_ block layer (bl) was deposited on FTO by a spin-coating step (3000 rpm, 30 s) and then annealed at 150°C for 1 h, named as p-SNO. SnO_2_ scaffold layer contained different Ln^3+^/Sn^4+^ molar ratios (0~4%) with thickness about 100~200 nm was covered by spin-coating the SnO_2_ solution at 2000 rpm for 30 s and then annealed at 300°C for 1 h to remove the organic residue, named, respectively, as m-SNO and *x*%-SNOLn (*x* = 1, 2, 3, 4). The cesium-containing triple cation perovskite was deposited by an antisolvent method according to the literatures [27, 60]. The perovskite precursor solution was spin-coated at 1000 rpm for 10 s and then at 6000 rpm for 20 s on the p-SNO and full SnO_2_ as bl/mp layers substrates. During the second step, 130 *μ*L of chlorobenzene was poured on the spinning substrate 5 s prior to the end of the program to rinse out residual DMSO and DMF in the precursor films. Afterwards, the substrates were heated immediately at 100 °C for 1 h and then were cooled down to room temperature naturally. Subsequently, the spiro-OMeTAD layers were subsequently deposited on top of the as-prepared perovskite layers by spin-coating 20 *μ*L of chlorobenzene solution containing chlorobenzene (1 mL), spiro-OMeTAD (80 mg), 4-tert-butylpyridine (28.8 *μ*L), and Li-TFSI (17.5 *μ*L, 520 mg Li-TFSI in 1 mL acetonitrile) at 4000 rpm for 30 s. Finally, about 100 nm thick Au electrodes were thermally evaporated on the spiro-OMeTAD layers under high vacuum via a shadow mask. Thus, the PSCs with the active area of 0.1 cm^2^ (0.25 × 0.4 cm^2^) were prepared. For clarity, PSC devices based on planar structure SnO_2_ and mesoporous structure SnO_2_ with different Ln^3+^/Sn^4+^ molar ratios (0~4%) are denoted as p-SNO device, m-SNO device, and *x*%-SNOLn devices (*x* = 1, 2, 3, 4). The PSCs with full TiO_2_ as bl/mp layers named as m-TiO_2_ were also fabricated as previously reported for comparative study [27].

### 4.4. Characterization

The crystal structures of samples were determined by powder X-ray diffraction (XRD, Smart Lab, Rigaku) using graphite monochromatic copper radiation (*λ* = 1.5418 *Å*). The morphology characterizations were performed on the field emission scanning electron microscopy (FE-SEM, SU8000, Hitachi) and a JEOL JEM-2100 transmission electron microscopy (TEM). Surface electronic states and UV photoelectron spectroscopy (UPS) were carried out using a XPS/UPS system (Thermo Scientific, ESCLAB 250XI, USA). UPS was performed using He I radiation at 21.22 eV with bias (−5 V) on the samples to separate the sample and analyzer low kinetic energy cutoffs. For XPS, all binding energies were referenced to the C1s peak (284.8 eV) of the surface adventitious. The Brunauer-Emmett-Teller (BET) specific surface area was determined using N_2_ adsorption apparatus (ASAP 2020 HD88, micromeritics) at 77 K after a pretreatment at 453 K for 3 h. The flat band potential was performed by using a CHI760E (Chenhua Co. Ltd, Shanghai) electrochemical workstation with a standard three-electrode configuration, which employed a Pt plate as the counter electrode and Ag/AgCl (saturated Na_2_SO_4_) as the reference electrode. Ultraviolet-visible (UV-vis) absorption spectra of samples were recorded on a PerkinElmer Lambda 950UV/VIS/NIR spectrometer in the wavelength range of 300~800 nm. The steady-state photoluminescence (PL) spectra were acquired using a fluorescence spectrophotometer (Lumina, Thermo Fisher) equipped with a Xenon lamp at an excitation wavelength of 507 nm. The time-resolved photoluminescence (TRPL) spectrum was recorded on an Omni-*λ* monochromator excited with a 760 nm laser.

### 4.5. Measurement

The photocurrent density-voltage (*J-V*) curves of PSCs were recorded with a Keithley 2420 source-measure unit under 100 mW cm^−2^ (AM 1.5G) with presweep delay of 0.04 s, max reverse bias of 0.2 V, max forward bias of 2.0 V, and dwell time of 30 ms in ambient environment. The illumination source was a solar light simulator (Newport Oriel Sol 3A class, USA, calibrated by a Newport reference cell). Average photovoltaic parameters of the PSC devices were obtained from 20 devices to ensure the reliability and repeatability of data. Dark *J-V* curves were measured on a Keithley 2420 source meter in the dark. The stabilized power output was recorded close to the maximum power point, which was extracted from the *J-V* curves on an electrochemical work station (CHI660E, Chenhua Co. Ltd, Shanghai) under simulated sunlight irradiation with intensity of 100 mW cm^−2^ at AM 1.5G. The incident photo-to-current conversion efficiency (IPCE) curves were measured as a function of wavelength from 300 nm to 800 nm using a QE-R quantum efficiency measurement system (Enli Technology Co. Ltd). The electrochemical impedance spectroscopy (EIS) measurements were conducted on a Zennium electrochemical workstation (IM6) under AM 1.5G with the frequencies from 100 mHz to 1 MHz, the bias of 0 V, and the amplitude of 20 mV. Long-term stability under persistent moisture (30% RH) was tested by XRD measurement for 7 days and record of time-dependent photovoltaic performances for 400 h under ambient condition with 30% RH. All the average values for long-term stability were obtained from 8 devices for each sample.

## Supplementary Materials

Supplementary Materials Figure S1: (a)–(c) survey XPS spectra of 3% Ln3+(Sc3+, Y3+, La3+)-doped m-SnO_2_. (d), (e) Narrow XPS spectra of Sc 2p and La 3d. Figure S2: (a) TEM and (b) HRTEM images of undoped m-SnO_2_. Figure S3: FE-SEM images of (a) m-SnO_2_, (b) 3% Sc3+, (c) 3% La3+, (d) 4% Sc3+, and (e) 4% La3+-doped m-SnO_2_. Top view FE-SEM images of (f) bare FTO glass and (g) p-SNO (annealed at 180°C). Figure S4: (a) I-V curves of various films under dark condition. (b) M-S plot of various films. The electrodes were submerged in a 0.5 M KCl solution with a Pt counter electrode and Ag/AgCl reference electrode. (c) PL spectra and (d) TRPL spectra of the perovskite films based on various SnO_2_ film. (e) Fitting curves from Nyquist plots for various SnO_2_ devices. The inset is the corresponding equivalent circuit. Figure S5: *J*-*V* curves of the PSCs based on m-SNO doped with (a) Sc3+, (b) Y 3+, and (c) La 3+ in different concentrations. (d)–(g) Average photovoltaic data of the devices with Ln-doped SnO_2_ scaffold. Average photovoltaic parameters of the each PSC device were obtained from 20 devices to ensure the reliability and repeatability of data. Figure S6: (a), (b) Dependence of JSC and VOC on light intensity of PSCs. (c) Dark *J*-*V* curves of PSCs. (d) *J*-*V* curves under dark conditions for the electron-only devices with the inserted structure. Table S1: pore properties of 3% Y-doped m-SnO_2_ and undoped m-SnO_2_. Table S2: band edge (Eg), Fermi level (EF), valence band (EVB), and conduction band (ECB) of samples. Table S3: fitted data from TRPL spectra in Figure S4d. Table S4: photovoltaic and impedance data of the PSCs. Table S5: PCE comparison of the PSCs based on full SnO_2_ mesoporous structure [S1-S8]. Table S6: photovoltaic data of the PSCs with different Ln3+ (Sc3+, Y3+, La3+) concentrations. Table S7: average photovoltaic data of the PSCs. The average values were obtained from 20 devices. Table S8: photovoltaic parameters of the PSCs scanning in different directions. Table S9: PCE values for 3%-SNOY and m-TiO_2_ devices. Average values were obtained from 8 devices.
