# Supplementary material for: High-Performance and Hysteresis-Free Perovskite Solar Cells Based on Rare-Earth-Doped SnO2 Mesoporous Scaffold
Source: Research (Wash D C). 2019 Nov 6;2019:4049793. doi: 10.34133/2019/4049793 (PMC6944519; doi:10.34133/2019/4049793)
Supplement: Supplementary Materials — Figure S1: (a)–(c) survey XPS spectra of 3% Ln3+(Sc3+, Y3+, La3+)-doped m-SnO2. (d), (e) Narrow XPS spectra of Sc 2p and La 3d. Figure S2: (a) TEM and (b) HRTEM images of undoped m-SnO2. Figure S3: FE-SEM images of (a) m-SnO2, (b) 3% Sc3+, (c) 3% La3+, (d) 4% Sc3+, and (e) 4% La3+-doped m-SnO2. Top view FE-SEM images of (f) bare FTO glass and (g) p-SNO (annealed at 180°C). Figure S4: (a) I-V curves of various films under dark condition. (b) M-S plot of various films. The electrodes were submerged in a 0.5 M KCl solution with a Pt counter electrode and Ag/AgCl reference electrode. (c) PL spectra and (d) TRPL spectra of the perovskite films based on various SnO2 film. (e) Fitting curves from Nyquist plots for various SnO2 devices. The inset is the corresponding equivalent circuit. Figure S5: J-V curves of the PSCs based on m-SNO doped with (a) Sc3+, (b) Y 3+, and (c) La 3+ in different concentrations. (d)–(g) Average photovoltaic data of the devices with Ln-doped SnO2 scaffold. Average photovoltaic parameters of the each PSC device were obtained from 20 devices to ensure the reliability and repeatability of data. Figure S6: (a), (b) Dependence of JSC and VOC on light intensity of PSCs. (c) Dark J-V curves of PSCs. (d) J-V curves under dark conditions for the electron-only devices with the inserted structure. Table S1: pore properties of 3% Y-doped m-SnO2 and undoped m-SnO2. Table S2: band edge (Eg), Fermi level (EF), valence band (EVB), and conduction band (ECB) of samples. Table S3: fitted data from TRPL spectra in Figure S4d. Table S4: photovoltaic and impedance data of the PSCs. Table S5: PCE comparison of the PSCs based on full SnO2 mesoporous structure [S1-S8]. Table S6: photovoltaic data of the PSCs with different Ln3+ (Sc3+, Y3+, La3+) concentrations. Table S7: average photovoltaic data of the PSCs. The average values were obtained from 20 devices. Table S8: photovoltaic parameters of the PSCs scanning in different directions. Table S9: PCE values for 3%-SNOY a [file 4049793.f1.doc]

**Supplementary Materials for**

**High Performance and Hysteresis-Free Perovskite Solar Cells Based on Rare-Earth Doped SnO_2_ Mesoporous Scaffold**

**Qiyao Guo^1^, Jihuai Wu^1^*,Yuqian Yang^1^, Xuping Liu^1^, Zhang Lan^1^, Jianming Lin^1^, Miaoliang Huang^1^, Yuelin Wei^1^, Jia Dong^2^, Jinbiao Jia^2^, and Yunfang Huang1,[[1]](#endnote-2)3**

^1^ Engineering Research Center of Environment-Friendly Functional Materials, Ministry of Education,

Institute of Materials Physical Chemistry, Huaqiao University, Xiamen, 361021, P. R. China.

^2^ School of Physics and Physical Engineering, Qufu Normal University, Qufu 273165, P. R. China.

^3^ School of Chemical Engineering, Huaqiao University, Xiamen, 361021, P. R. China.

Correspondence should be addressed to Jihuai WU; [jhwu@hqu.edu.cn](mailto:jhwu@hqu.edu.cn)

**This PDF file includes:**

**Figures S1~S6**

**Tables S1~S9**

**References S1~S8**

**
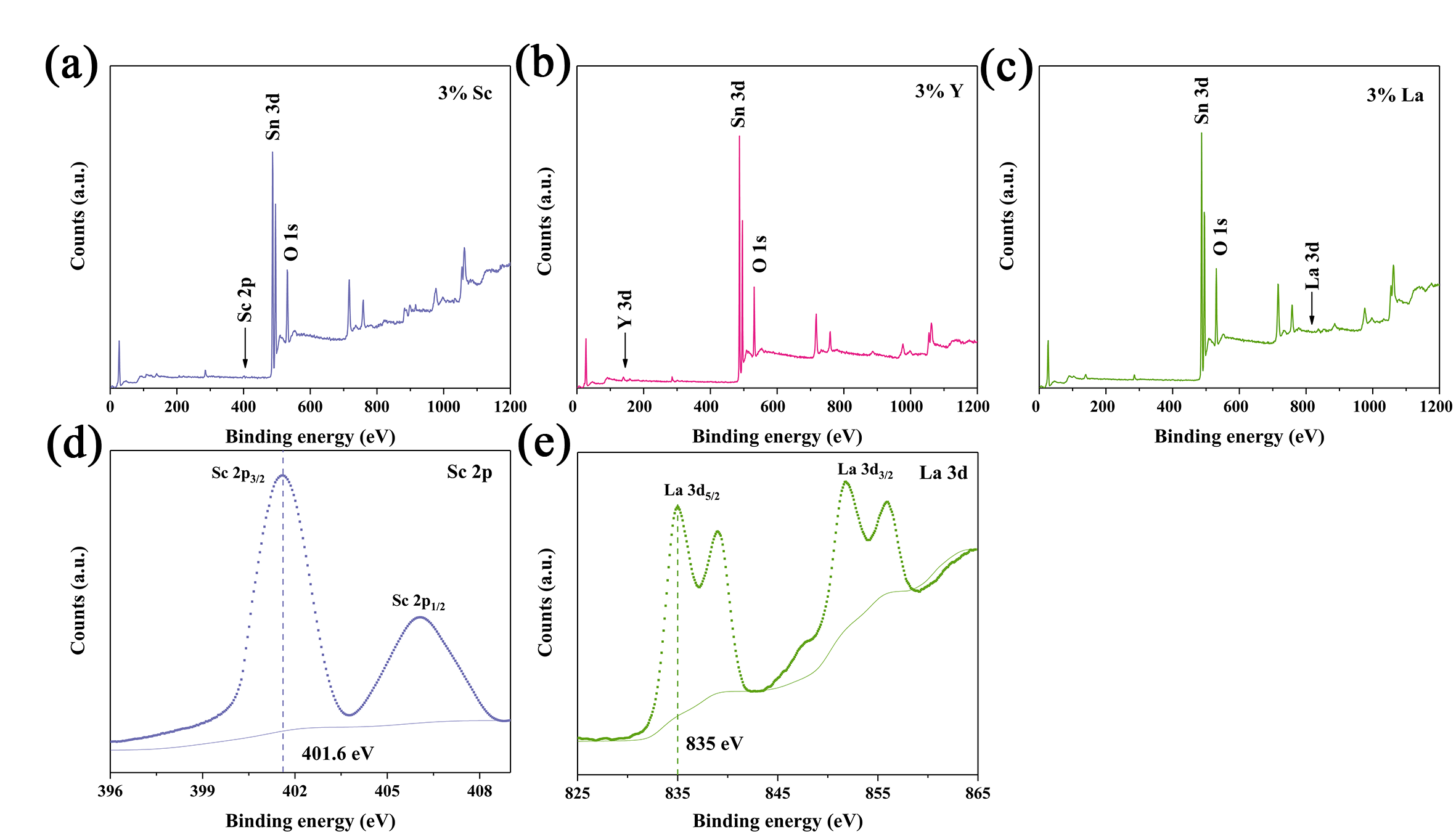
**

Figure S1: (a)~(c) Survey XPS spectra of 3% Ln^3+^(Sc^3+^, Y^3+^, La^3+^) doped m-SnO_2_. (d), (e) Narrow XPS spectra of Sc 2p and La 3d.


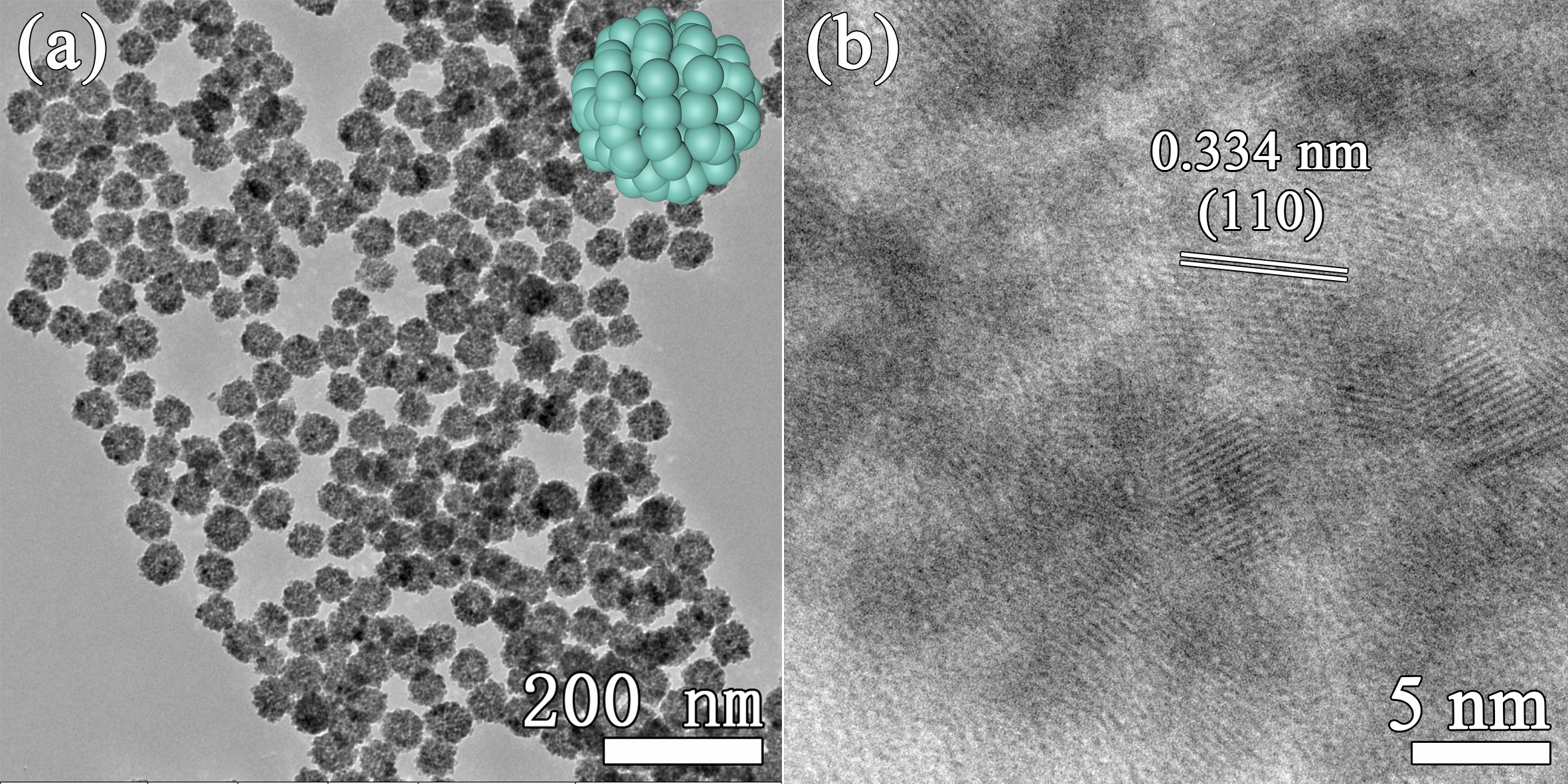


Figure S2: (a) TEM and (b) HRTEM images of undoped m-SnO_2_.


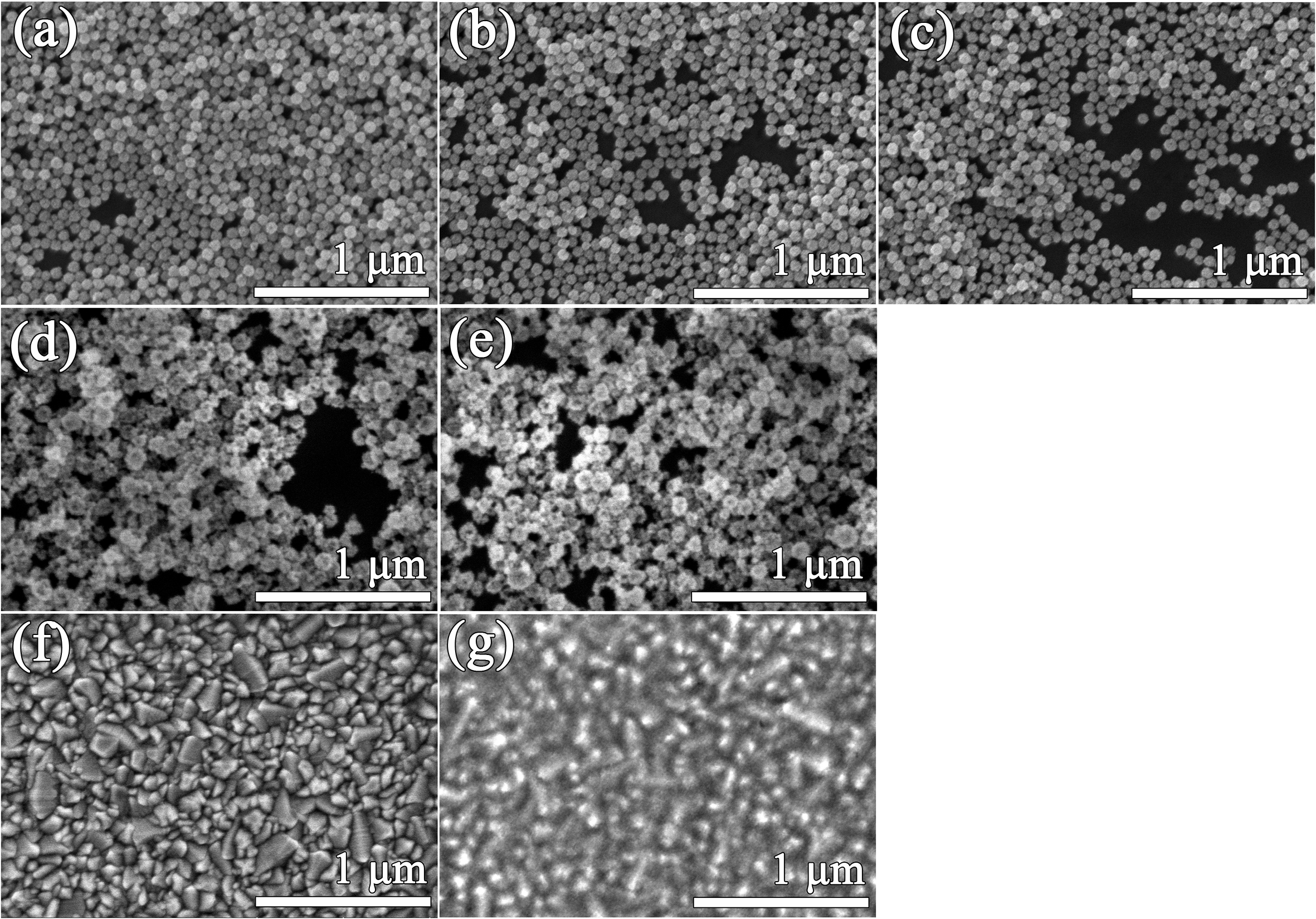


Figure S3: FE-SEM images of (a) m-SnO_2_, (b) 3% Sc^3+^, (c) 3% La^3+^, (d) 4% Sc^3+^, (e) 4% La^3+^ doped m-SnO_2_ films. Top view FE-SEM images of (f) bare FTO glass, (g) p-SNO (annealed at 180 °C).


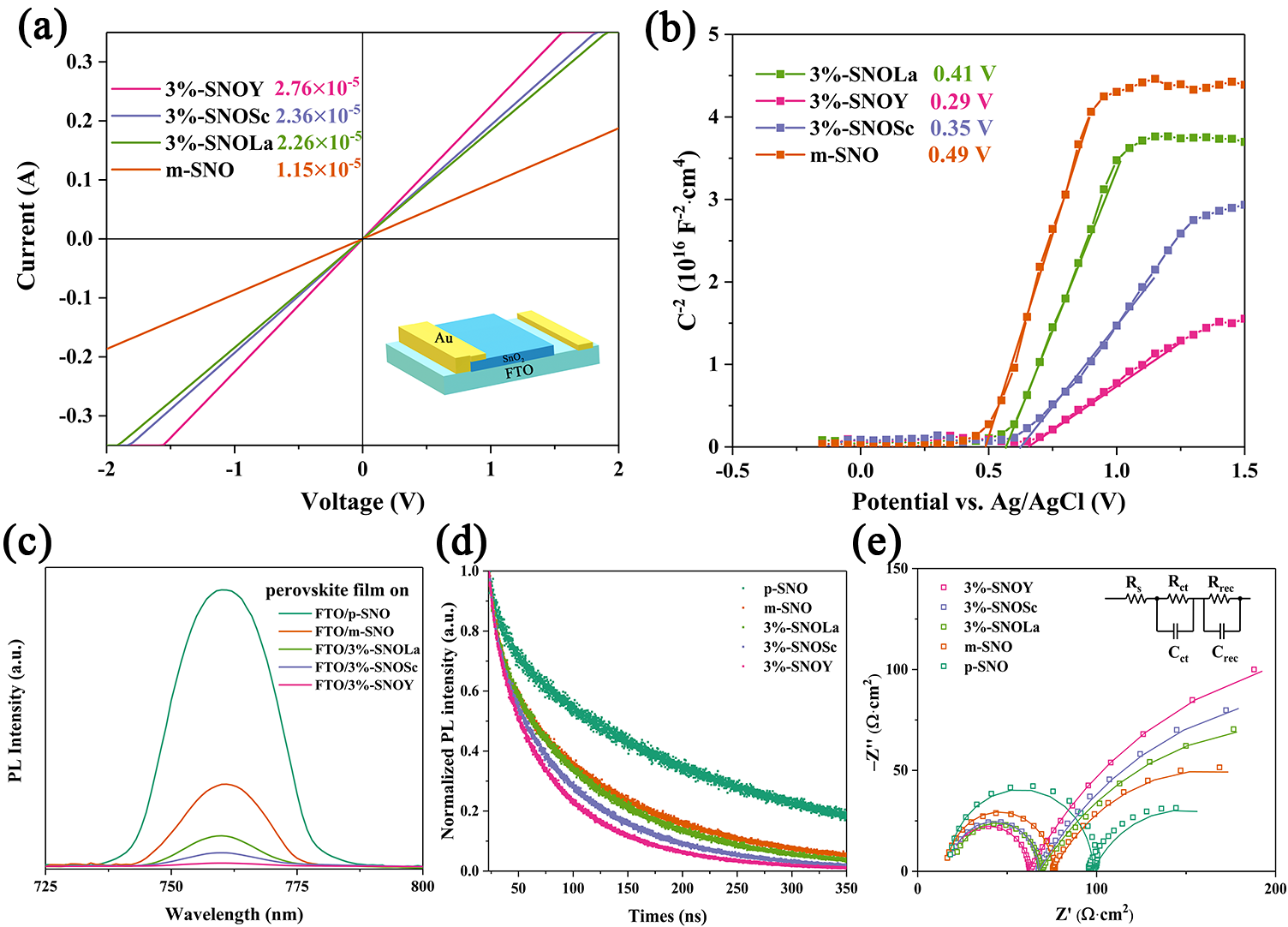


Figure S4: (a) *I-V* curves of various ﬁlms under dark condition. (b) M–S plot of various ﬁlms. The electrodes were submerged in a 0.5 M KCl solution with a Pt counter electrode and Ag/AgCl reference electrode. (c) PL spectra, (d) TRPL spectra of the perovskite ﬁlms based on various SnO_2_ film. (e) Fitting curves from Nyquist plots for various SnO_2_ devices. The inset is the corresponding equivalent circuit.


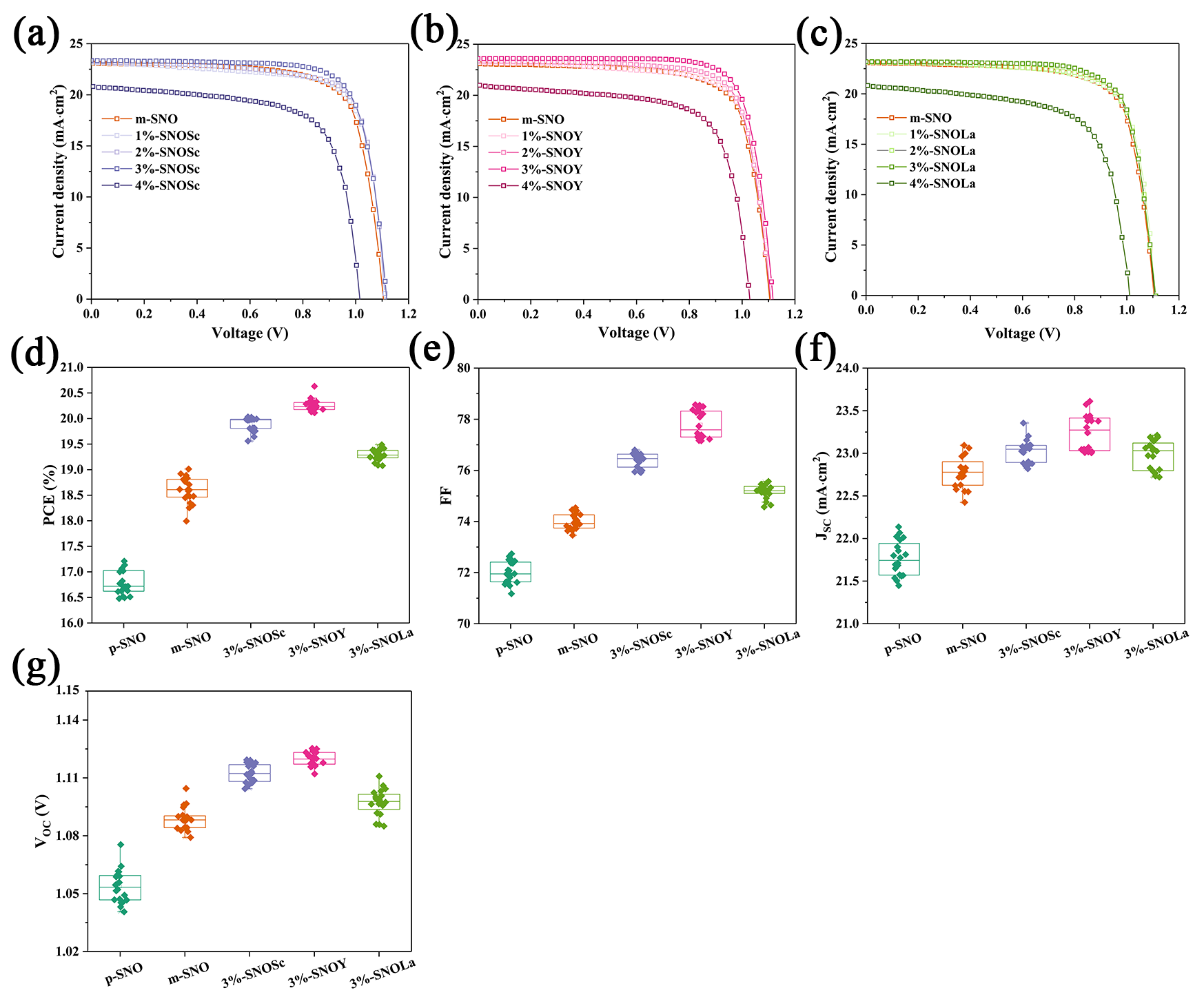


Figure S5: *J-V* curves of the PSCs based on m-SNO doped with (a) Sc^3+^, (b) Y ^3+^, (c) La ^3+^ in different concentrations. (d)~(g) Average photovoltaic data of the devices with Ln doped SnO_2_ scaffold. Average photovoltaic parameters of the each PSC device were obtained from 20 devices to ensure the reliability and repeatability of data.


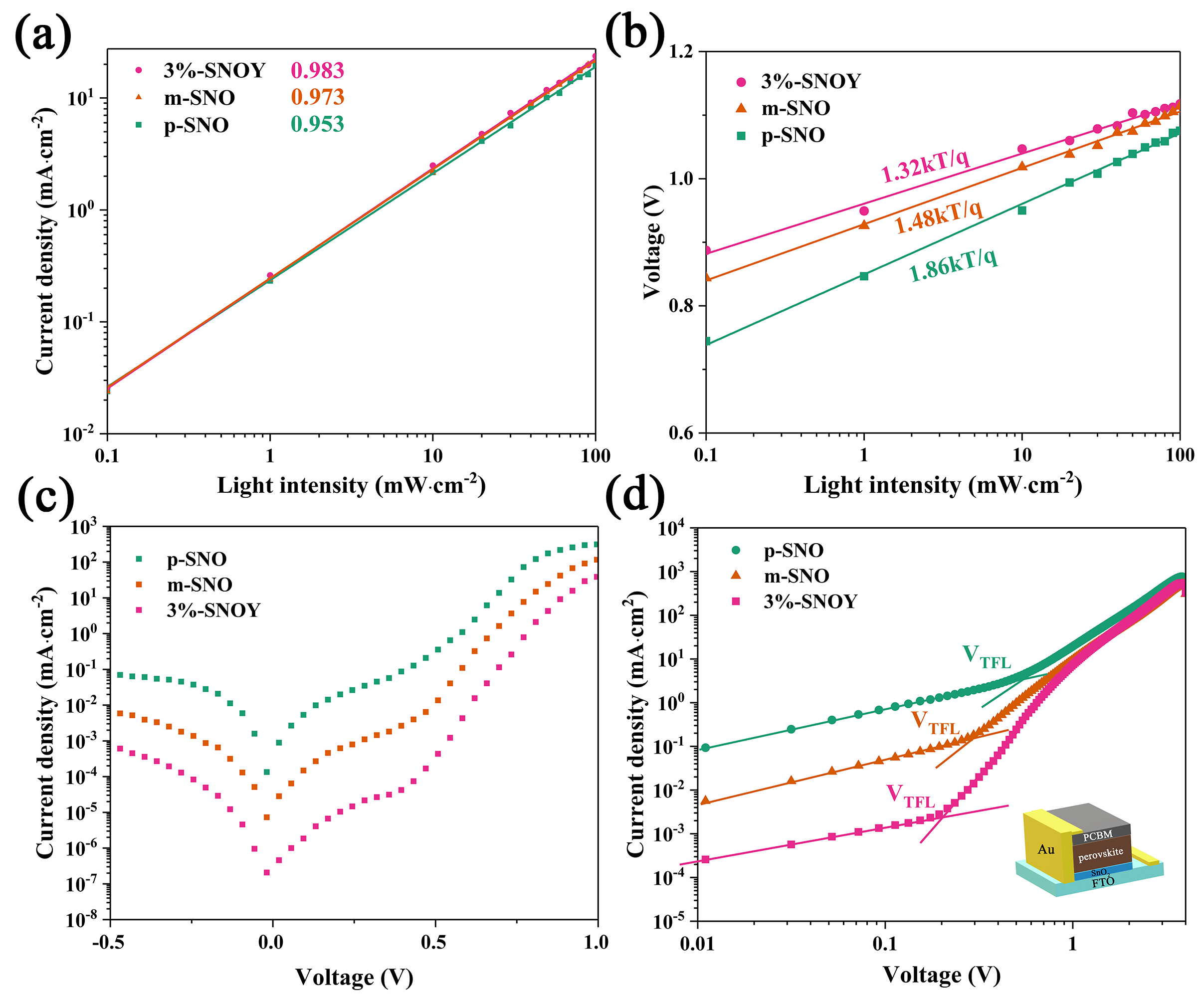


Figure S6: (a), (b) Dependence of *J*_SC_ and *V*_OC_ on light intensity of PSCs. (c) Dark *J*-*V* curves of PSCs. (d) *J*-*V* curves under dark conditions for the electron-only devices with the inserted structure.

| Table S1: Pore properties of 3% Y doped m-SnO_2_ and undoped m-SnO_2_. | | |
| --- | --- | --- |
| Sample | S_BET_^a^ (m^2^·g^–1^) | Pore width (nm) |
| 3% Y doped m-SnO_2_ | 130.0 | 13.8 |
| undoped m-SnO_2_ | 120.2 | 9.80 |

^a^ S_BET_ is the speciﬁc surface area.

| Table S2: Band edge (*E*_g_), Fermi level (*E*_F_), valence band (*E*_VB_) and conduction band (*E*_CB_) of samples. | | | | |
| --- | --- | --- | --- | --- |
| Sample | *E*_g_ (eV) | *E*_F_ (eV) | *E*_VB_ (eV) | *E*_CB_ (eV) |
| p-SNO | 3.95 | −5.37 | −8.44 | −4.49 |
| m-SNO | 3.95 | −5.18 | −8.26 | −4.31 |
| 3%-SNOSc | 3.95 | −5.03 | −8.14 | −4.19 |
| 3%-SNOY | 3.95 | −5.01 | −8.09 | −4.13 |
| 3%-SNOLa | 3.96 | −5.09 | −8.20 | −4.25 |

| Table S3: Fitted data from TRPL spectra in Figure S4d. | | | | | |
| --- | --- | --- | --- | --- | --- |
| Sample | τ_ave_ (ns) | τ_1_ (ns) | τ_2_ (ns) | *A*_1_ (%) | *A_2_* (%) |
| p-SNO | 171.35 | 202.78 | 16.17 | 83.16 | 16.84 |
| m-SNO | 89.42 | 119.14 | 15.26 | 71.40 | 28.60 |
| 3%-SNOSc | 70.31 | 87.71 | 16.86 | 75.44 | 24.56 |
| 3%-SNOY | 58.89 | 76.54 | 20.42 | 68.55 | 31.45 |
| 3%-SNOLa | 83.08 | 104.38 | 14.77 | 76.23 | 23.77 |

| Table S4: Photovoltaic and impedance data of the PSCs. | | | | | | | |
| --- | --- | --- | --- | --- | --- | --- | --- |
| Sample | *J*_SC_ (mA·cm^−2^) | *V*_OC_ (V) | FF (%) | PCE (%) | *R*_s_ (Ω) | *R*_ct_ (Ω) | *R*_rec_ (Ω) |
| p-SNO | 22.06 | 1.075 | 72.51 | 17.21 | 16.90 | 104.4 | 81.06 |
| m-SNO | 23.09 | 1.105 | 74.54 | 19.01 | 16.16 | 59.37 | 173.3 |
| 3%-SNOSc | 23.36 | 1.116 | 76.80 | 20.03 | 18.14 | 49.39 | 314.6 |
| 3%-SNOY | 23.61 | 1.117 | 78.16 | 20.63 | 17.46 | 44.65 | 380.7 |
| 3%-SNOLa | 23.21 | 1.111 | 75.57 | 19.49 | 20.45 | 47.87 | 269.8 |

| Table S5: PCE comparison of the PSCs based on full SnO_2_ mesoporous structure [S1-S8]. | | | | |
| --- | --- | --- | --- | --- |
| Ref. | Annealing temperature (°C) | Year | Champion PCE (%) | |
| [S1] | 500 | 2015 | | 10.18 |
| [S2] | low-temperature approach | 2016 | | 16.17 |
| [S3] | 450 | 2016 | | 13.10 |
| [S4] | low-temperature approach | 2017 | | 17.29 |
| [S5] | 450 | 2018 | | 17.00 |
| [S6] | 500 | 2018 | | 19.21 |
| [S7] | 450 | 2019 | | 13.00 |
| [S8] | 450 | 2019 | | 15.41 |
| Our work | 300 | – | | 20.63 |

| Table S6: Photovoltaic data of the PSCs with different Ln^3+^ (Sc^3+^, Y^3+^, La^3+^) concentrations. | | | | | | | | | |
| --- | --- | --- | --- | --- | --- | --- | --- | --- | --- |
| Sample | *J*_SC_ (mA/cm^2^) | | | *V*_OC_ (V) | | FF (%) | | PCE (%) | |
| m-SNO | | 23.09 | | 1.105 | | 74.54 | | 19.01 | |
| 1%-SNOSc | | 23.12 | | 1.108 | | 75.03 | | 19.19 | |
| 2%-SNOSc | | 23.24 | | 1.113 | | 75.64 | | 19.56 | |
| 3%-SNOSc | | | 23.36 | | 1.117 | | 76.80 | | 20.03 |
| 4%-SNOSc | | 20.87 | | 1.016 | | 69.05 | | 14.64 | |
| 1%-SNOY | | 23.21 | | 1.109 | | 74.94 | | 19.30 | |
| 2%-SNOY | | 23.35 | | 1.112 | | 76.56 | | 19.81 | |
| 3%-SNOY | | 23.61 | | 1.117 | | 78.16 | | 20.63 | |
| 4%-SNOY | | 21.02 | | 1.029 | | 70.03 | | 15.15 | |
| 1%-SNOLa | | 23.11 | | 1.106 | | 74.79 | | 19.13 | |
| 2%-SNOLa | | 23.16 | | 1.107 | | 75.15 | | 19.28 | |
| 3%-SNOLa | | 23.21 | | 1.111 | | 75.57 | | 19.49 | |
| 4%-SNOLa | | 20.88 | | 1.011 | | 67.22 | | 14.19 | |

| Table S7: Average photovoltaic data of the PSCs. The average values were obtained from 20 devices. | | | | |
| --- | --- | --- | --- | --- |
| Sample | *J*_SC_ (mA/cm^2^) | *V*_OC_ (V) | FF (%) | PCE (%) |
| p-SNO | 21.76 ± 0.206 | 1.054 ± 0.009 | 72.01 ± 0.440 | 16.79 ± 0.233 |
| m-SNO | 22.77 ± 0.183 | 1.089 ± 0.006 | 74.00 ± 0.311 | 18.60 ± 0.256 |
| 3%-SNOSc | 23.03 ± 0.133 | 1.112 ± 0.005 | 76.39 ± 0.281 | 19.89 ± 0.138 |
| 3%-SNOY | 23.24 ± 0.211 | 1.120 ± 0.004 | 77.80 ± 0.546 | 20.26 ± 0.116 |
| 3%-SNOLa | 22.98 ± 0.170 | 1.096 ± 0.006 | 75.17 ± 0.274 | 19.28 ± 0.109 |

| Table S8: Photovoltaic parameters of the PSCs scanning in different directions. | | | | | | |
| --- | --- | --- | --- | --- | --- | --- |
| Devices | Scanning direction | | *J*_SC_ (mA·cm^−2^) | *V*_OC_ (V) | FF (%) | PCE (%) |
| p-SNO | Forward | 22.01 | | 1.045 | 70.38 | 16.54 |
|  | Backward | 22.06 | | 1.075 | 72.51 | 17.21 |
| m-SNO | Forward | 23.08 | | 1.089 | 74.08 | 18.60 |
|  | Backward | 23.09 | | 1.105 | 74.54 | 19.01 |
| 3%-SNOY | Forward | 23.61 | | 1.115 | 77.34 | 20.40 |
|  | Backward | 23.61 | | 1.117 | 78.16 | 20.63 |

| Table S9: PCE values of 3%-SNOY and m-TiO_2_ devices. Average values were obtained from 8 devices. | | | | | | |
| --- | --- | --- | --- | --- | --- | --- |
| Time (h) | 3%-SNOY | m-TiO_2_ | Time (h) | | 3%-SNOY | m-TiO_2_ |
| 0 | 20.26 ± 0.116 | 18.15 ± 0.181 | | 240 | 16.65 ± 0.292 | 10.18 ± 0.265 |
| 40 | 18.88 ± 0.151 | 11.76 ± 0.194 | | 280 | 16.53 ± 0.361 | 9.728 ± 0.448 |
| 80 | 18.23 ± 0.194 | 11.54 ± 0.332 | | 320 | 16.29 ± 0.245 | 9.874 ± 0.325 |
| 120 | 17.63 ± 0.176 | 10.69 ± 0.352 | | 360 | 15.68 ± 0.219 | 9.656 ± 0.414 |
| 160 | 17.44 ± 0.217 | 10.29 ± 0.298 | | 400 | 15.36 ± 0.261 | 9.656 ± 0.307 |
| 200 | 17.10 ± 0.304 | 10.35 ± 0.430 | |  |  |  |

**References**

[S1] Y. Li, J. Zhu, Y. Huang et al., "Mesoporous SnO_2_ nanoparticle films as electron-transporting material in perovskite solar cells," *RSC Adv.*, vol. 5, no. 36, pp. 28424–28429, 2015.

[S2] L. B. Xiong, M. C. Qin, G. Yang et al., "Performance enhancement of high temperature SnO_2_-based planar perovskite solar cells: electrical characterization and understanding of the mechanism," *Journal of Materials Chemistry A*, vol. 4, no. 21, pp. 8374–8383, 2016.

[S3] B. Roose, J.-P. C. Baena, K. C. Gödel et al., "Mesoporous SnO_2_ electron selective contact enables UV-stable perovskite solar cells," *Nano Energy*, vol. 30, pp. 517–522, 2016.

[S4] G. Yang, H. W. Lei, H. Tao et al., "Reducing Hysteresis and Enhancing Performance of Perovskite Solar Cells Using Low-Temperature Processed Y-Doped SnO_2_ Nanosheets as Electron Selective Layers," Small, vol. 13, no. 2, pp. 1601769, 2017.

[S5] B. Roose, C. M. Johansen, K. Dupraz et al., "A Ga-doped SnO_2_ mesoporous contact for UV stable highly efficient perovskite solar cells," *Journal of Materials Chemistry A*, vol. 6, no. 4, pp. 1850–1857, 2018.

[S6] L. B. Xiong, M. C. Qin, C. Chen et al., "Fully High-Temperature-Processed SnO_2_ as Blocking Layer and Scaffold for Efficient, Stable, and Hysteresis-Free Mesoporous Perovskite Solar Cells," *Advanced Functional Materials*, vol. 28, no. 10, pp. 1706276, 2018.

[S7] B. Roose, K. C. Gödel, S. Pathak et al., "Enhanced Efficiency and Stability of Perovskite Solar Cells Through Nd-Doping of Mesostructured TiO_2_," *Advanced Energy Materials*, vol. 6, no. 2, pp. 1501868, 2016.

[S8] X. Zhang, Y. Rui, J. Yang et al., "Monodispersed SnO_2_ microspheres aggregated by tunable building units as effective photoelectrodes in solar cells," *Applied Surface Science*, vol. 463, pp. 679–685, 2019.

1. ^3^ [↑](#endnote-ref-2)
